# Supplementary material for: Molecular and functional profiling unravels targetable vulnerabilities in colorectal cancer
Source: Mol Oncol. 2025 Jan 28;19(6):1751–74. doi: 10.1002/1878-0261.13814 (PMC12161475; doi:10.1002/1878-0261.13814)
Supplement: Supplementary file 1 — Fig. S1. Relative contribution of COSMIC mutational signatures in each patient of the Athens Comprehensive Cancer Center Colorectal Cancer (ACCC‐CRC) cohort. [file MOL2-19-1751-s008.pdf]

Supplementary Fig. 1

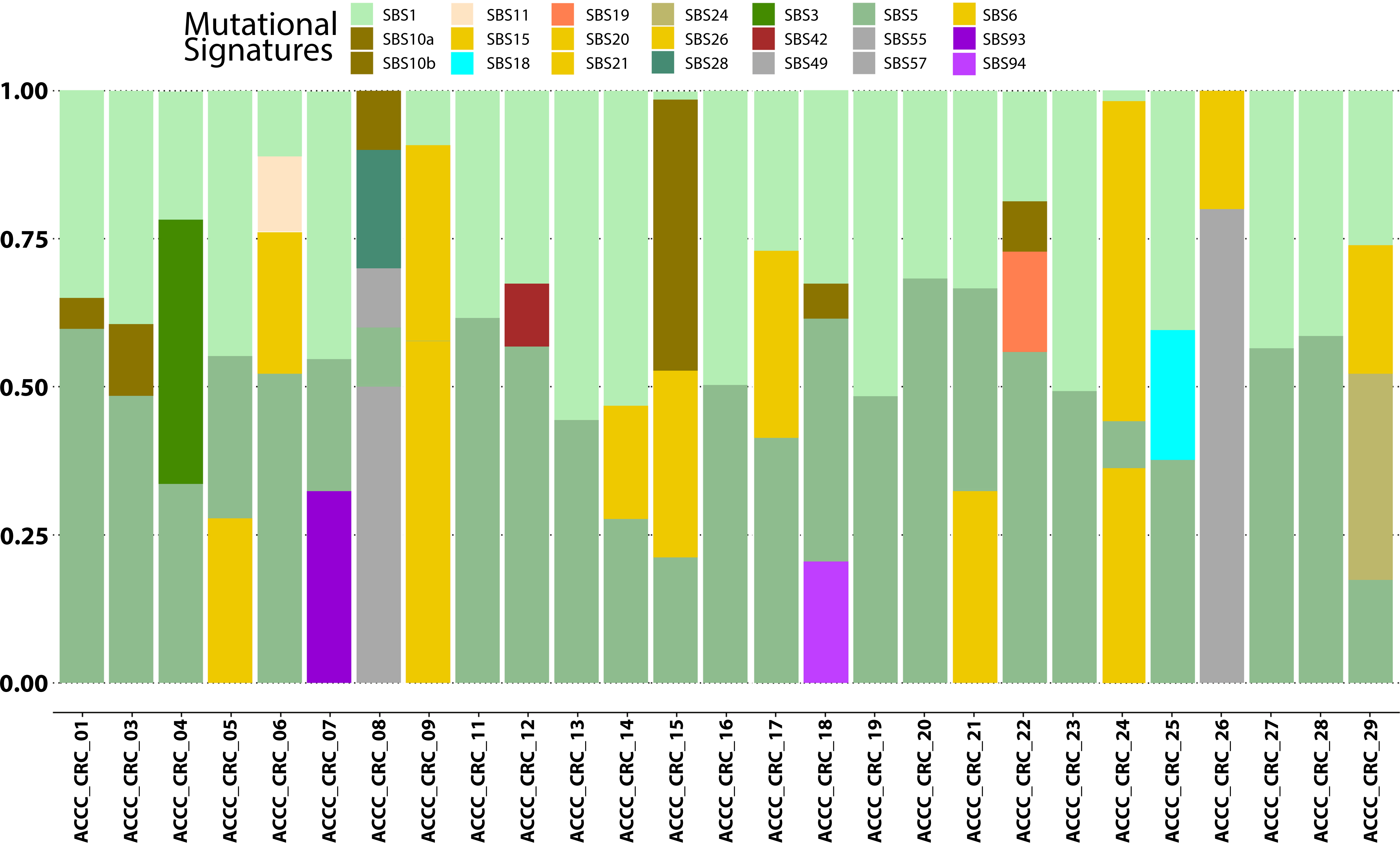

Relative contribution of COSMIC mutational signatures in each patient of the Athens Comprehensive Cancer Center Colorectal Cancer (ACCC-CRC) cohort. Single base substitution (SBS) signatures [23] were assessed using the SigProfilerAssignment web tool and the ggplot2 R package (v.3.4.3). Signatures accounting for similar biological processes were grouped together into the same "color" representation in the barplot, based on the following scheme: microsatellite instable (MSI)-related signatures [SBS6, SBS15, SBS20, SBS26] and POLE-related mutational procedures [SBS10a and SBS10b].
