# Supplementary figures and images for: Molecular and functional profiling unravels targetable vulnerabilities in colorectal cancer
Source: Mol Oncol. 2025 Jan 28;19(6):1751–74. doi: 10.1002/1878-0261.13814 (PMC12161475; doi:10.1002/1878-0261.13814)

Supplementary Fig. 2

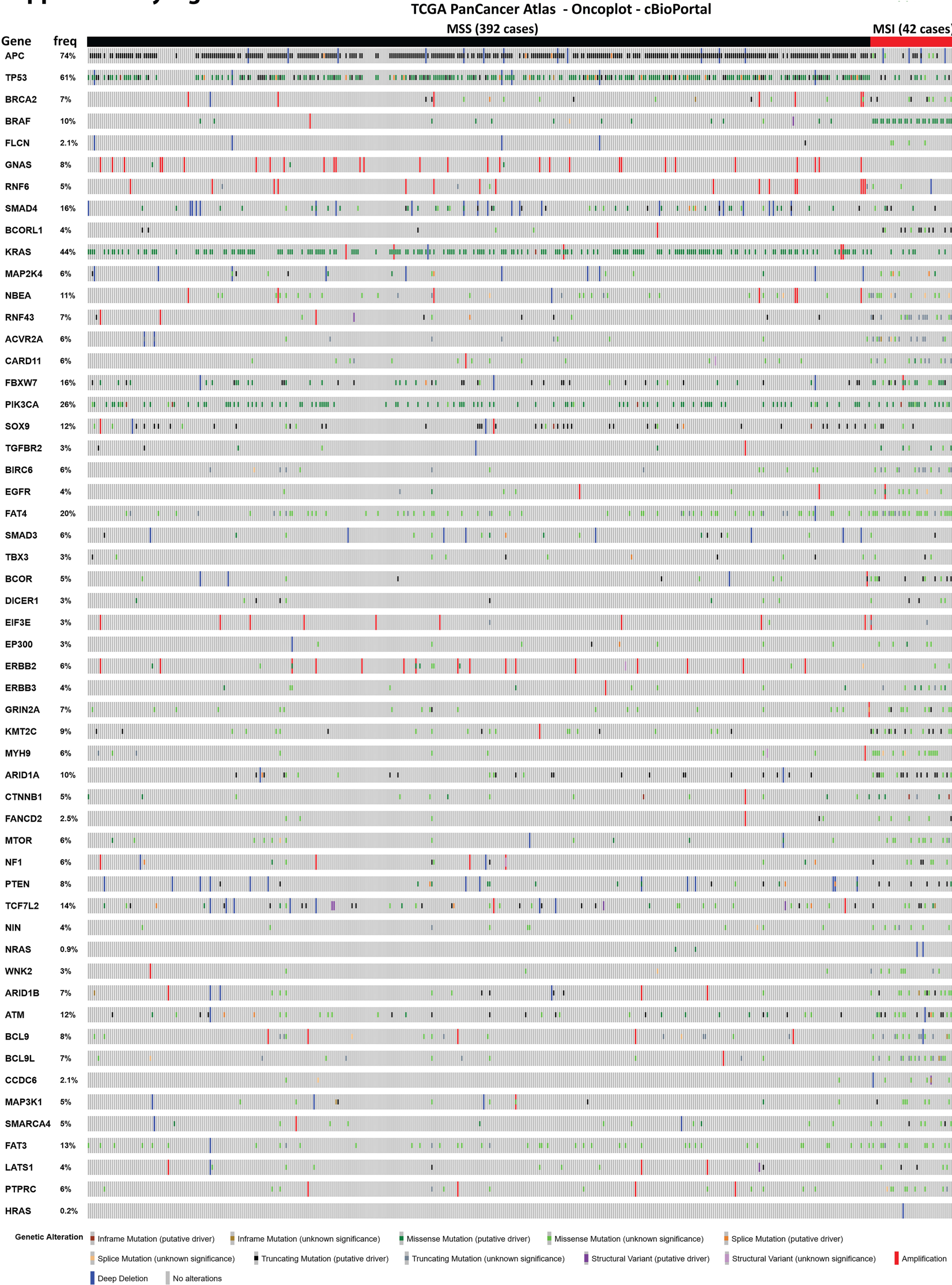

Supplement: Supplementary file 2 — Fig. S2. Oncoplot of 54 cancer driver genes across 434 colorectal adenocarcinoma samples from The Cancer Genome Atlas Network (TCGA) PanCancer Atlas cohort (392 microsatellite stable‐MSS and 42 microsatellite instable‐MSI cases). [file MOL2-19-1751-s010.pdf]
