# Supplementary material for: Molecular and functional profiling unravels targetable vulnerabilities in colorectal cancer
Source: Mol Oncol. 2025 Jan 28;19(6):1751–74. doi: 10.1002/1878-0261.13814 (PMC12161475; doi:10.1002/1878-0261.13814)
Supplement: Supplementary file 3 — Fig. S3. Oncoplot of 54 cancer driver genes across 79 colorectal adenocarcinoma samples from the National Cancer Institute's Clinical Proteomic Tumor Analysis Consortium (CPTAC) cohort (65 microsatellite stable‐MSS and 14 microsatellite instable‐MSI cases). [file MOL2-19-1751-s011.pdf]

Supplementary Fig. 3

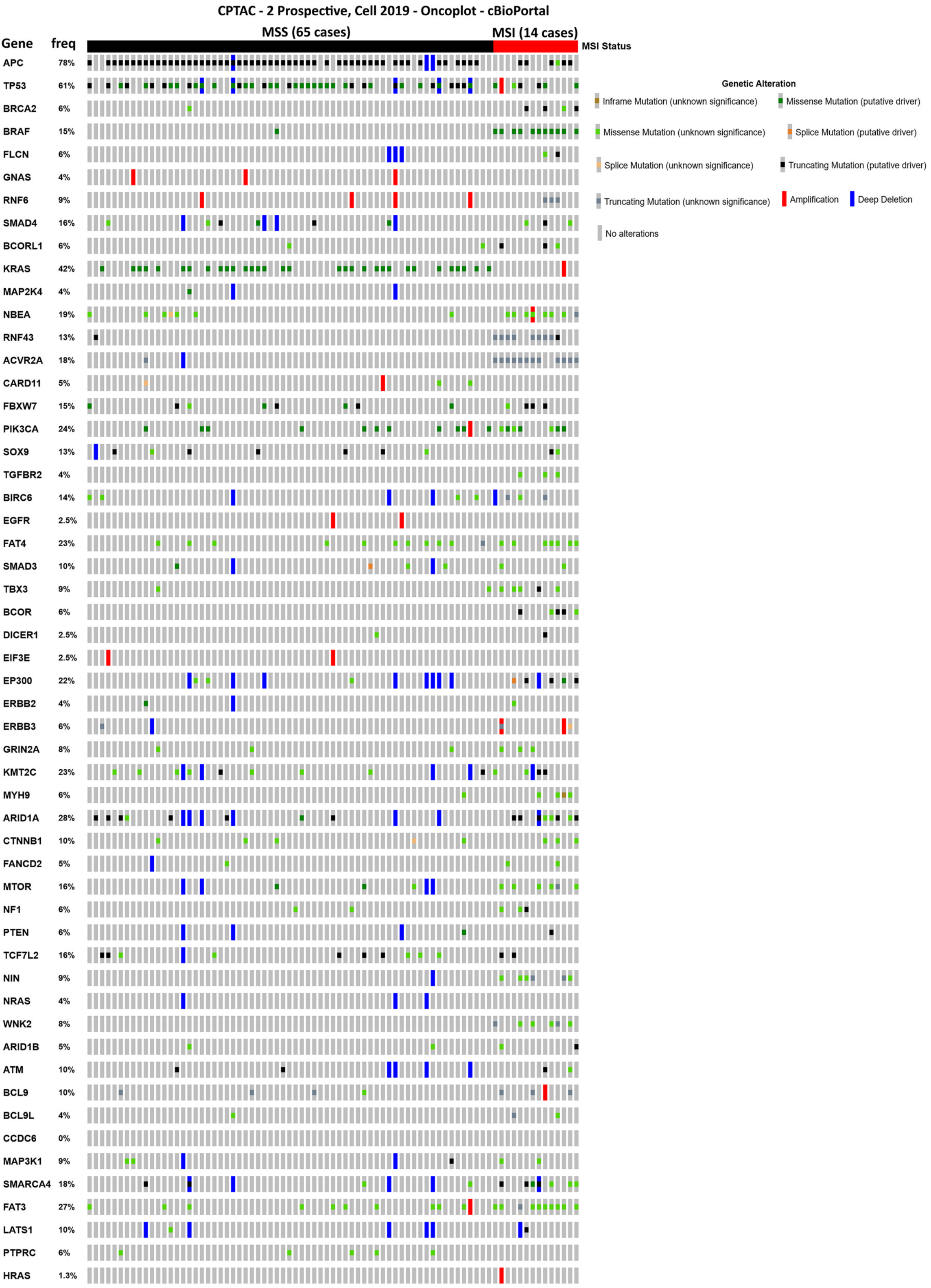

Oncoplot of 54 cancer driver genes across 79 colorectal adenocarcinoma samples from the National Cancer Institute's Clinical Proteomic Tumor Analysis Consortium (CPTAC) cohort (65 microsatellite stable-MSS and 14 microsatellite instable-MSI cases).
The oncoplot (produced in cBioPortal) shows the somatic mutation landscape of colorectal cancer (CRC) tumors including somatic mutations, structural variants and putative copy-number alterations identified using GISTIC 2.0. Each column represents a single tumor sample, while each row corresponds to one of the 54 selected cancer driver genes. For each gene, the alteration frequency (freq) is also displayed as the percentage of altered samples relative to the total number of profiled samples. The types of genetic alterations shown include missense mutations, truncating mutations, in-frame mutations, splice mutations, amplifications, and deep deletions. The color-coded bar at the right side of the oncoplot indicates the different categories of genetic alterations.
