# Supplementary material for: Molecular and functional profiling unravels targetable vulnerabilities in colorectal cancer
Source: Mol Oncol. 2025 Jan 28;19(6):1751–74. doi: 10.1002/1878-0261.13814 (PMC12161475; doi:10.1002/1878-0261.13814)
Supplement: Supplementary file 4 — Fig. S4. K‐means clustering of STRING protein–protein interaction network of high mutational frequency genes in microsatellite instable (MSI) vs. microsatellite stable (MSS) colorectal cancer. [file MOL2-19-1751-s013.pdf]

Supplementary Fig. 4

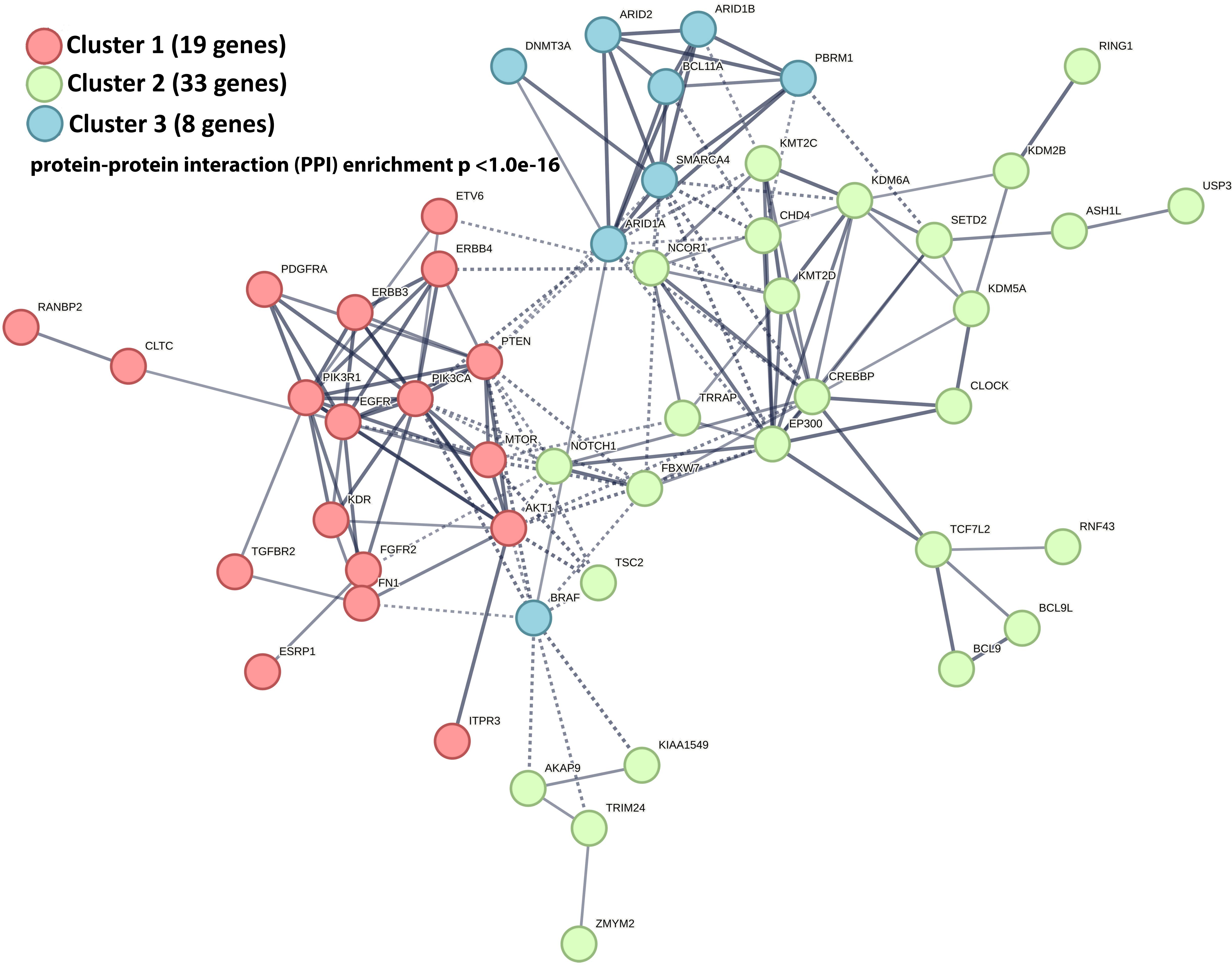

K-means clustering of STRING protein-protein interaction network of high mutational frequency genes in microsatellite instable (MSI) vs. microsatellite stable (MSS) colorectal cancer. The network was generated using the STRING database (version 12.0) with an input of 60 genes identified to have a higher mutational frequency in MSI compared to MSS colorectal cancer (CRC) tumors. Interactions were considered at a confidence threshold score ≥0.7. The network was clustered into groups using k-means clustering. Respective genes within clusters are color coded. Solid lines indicate direct functional or/and physical protein associations within clusters, while dotted lines represent interactions between clusters. Gene symbols are indicated in the network.
