# Supplementary material for: Molecular and functional profiling unravels targetable vulnerabilities in colorectal cancer
Source: Mol Oncol. 2025 Jan 28;19(6):1751–74. doi: 10.1002/1878-0261.13814 (PMC12161475; doi:10.1002/1878-0261.13814)
Supplement: Supplementary file 5 — Fig. S5. Kinase and Pathway activities, and GNAS alterations in microsatellite stable (MSS) and microsatellite instable (MSI) colorectal cancer (CRC) tumors of the National Cancer Institute's Clinical Proteomic Tumor Analysis Consortium (CPTAC) cohort. [file MOL2-19-1751-s004.pdf]

Supplementary Fig. 5

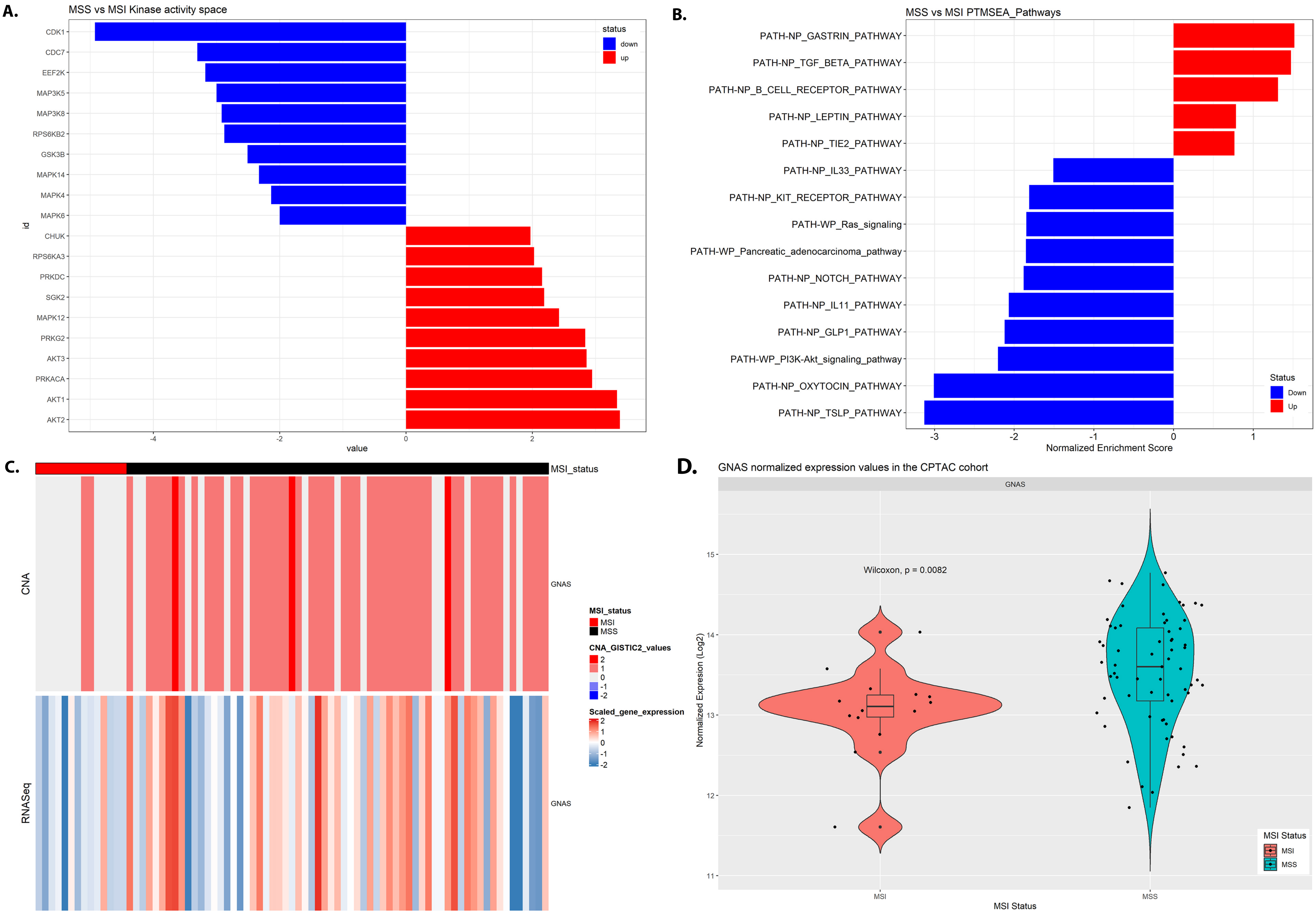

Kinase and Pathway activities, and GNAS alterations in microsatellite stable (MSS) and microsatellite instable (MSI) colorectal cancer (CRC) tumors of the National Cancer Institute's Clinical Proteomic Tumor Analysis Consortium (CPTAC) cohort. (A) Barplot of the top-10 most activated (red bars) and most repressed (blue bars) kinases between MSS and MSI colorectal cancer (CRC) tumors, based on differential phospho-proteomic analysis from the CPTAC patient cohort, with normalized enrichment scores (NES) from the OmnipathR-decoupleR pipeline [55]. Inferred kinase activities are indicated in red (higher in MSS, NES ≥2) and blue (higher in MSI, NES ≤-2) colors, respectively. (B) Barplot of estimated pathway activities, showing top-selected activated (red) and repressed (blue) pathways, with NES derived from PTM-SEA [57] using PTMSigDB pathway collection (version 2.0.0). (C) Heatmap of GNAS copy number alterations (from GISTIC 2.0) alongside scaled gene expression values (RNAseq data) of CPTAC-CRC tumors, stratified by their MSI status. (D) Violin plot detailing GNAS normalized expression values between MSS and MSI tumors of CPTAC cohort.
