# Supplementary material for: Molecular and functional profiling unravels targetable vulnerabilities in colorectal cancer
Source: Mol Oncol. 2025 Jan 28;19(6):1751–74. doi: 10.1002/1878-0261.13814 (PMC12161475; doi:10.1002/1878-0261.13814)
Supplement: Supplementary file 6 — Fig. S6. Clustering of the colorectal cancer (CRC) patients of The Cancer Genome Atlas Network (TCGA) based on transcriptomic analysis. [file MOL2-19-1751-s003.pdf]

Supplementary Fig. 6

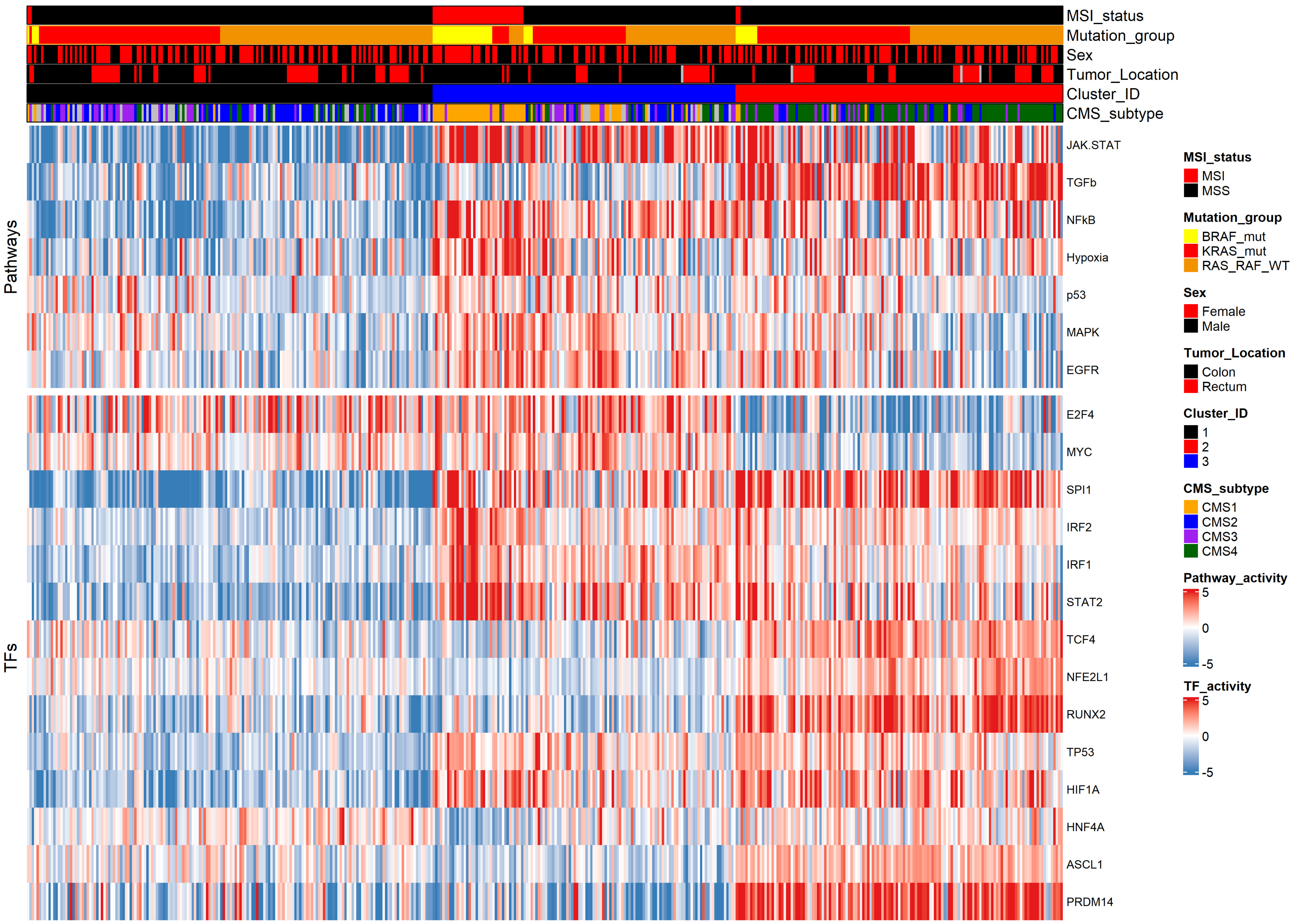

Clustering of the colorectal cancer (CRC) patients of The Cancer Genome Atlas Network (TCGA) based on transcriptomic analysis. Normalized enrichment scores (NES) for the common 7 pathways and 14 transcription factor (TF) activities (refer to Fig. 3) were utilized for unsupervised clustering on principal components (HCPC) using the R packages FactoMineR (version 2.8) and Factoshiny (version 2.4; http://factominer.free.fr/graphs/factoshiny.html), categorizing TCGA CRC patients into 3 distinct clusters. Features within each pathway and TF sub-cluster were re-ordered using hierarchical clustering ("complete" method, "euclidean" distance), first based on the cluster ID and subsequently by microsatellite instability (MSI) status and mutational profiles. NES values for pathway and TF activities are represented as red (high activity) or blue (low activity) gradients.
