# Supplementary material for: Molecular and functional profiling unravels targetable vulnerabilities in colorectal cancer
Source: Mol Oncol. 2025 Jan 28;19(6):1751–74. doi: 10.1002/1878-0261.13814 (PMC12161475; doi:10.1002/1878-0261.13814)
Supplement: Supplementary file 7 — Fig. S7. Clustering of the National Cancer Institute's Clinical Proteomic Tumor Analysis Consortium Colon Adenocarcinoma (CPTAC‐COAD) dataset based on transcriptomic profiling. [file MOL2-19-1751-s002.pdf]

Supplementary Fig. 7

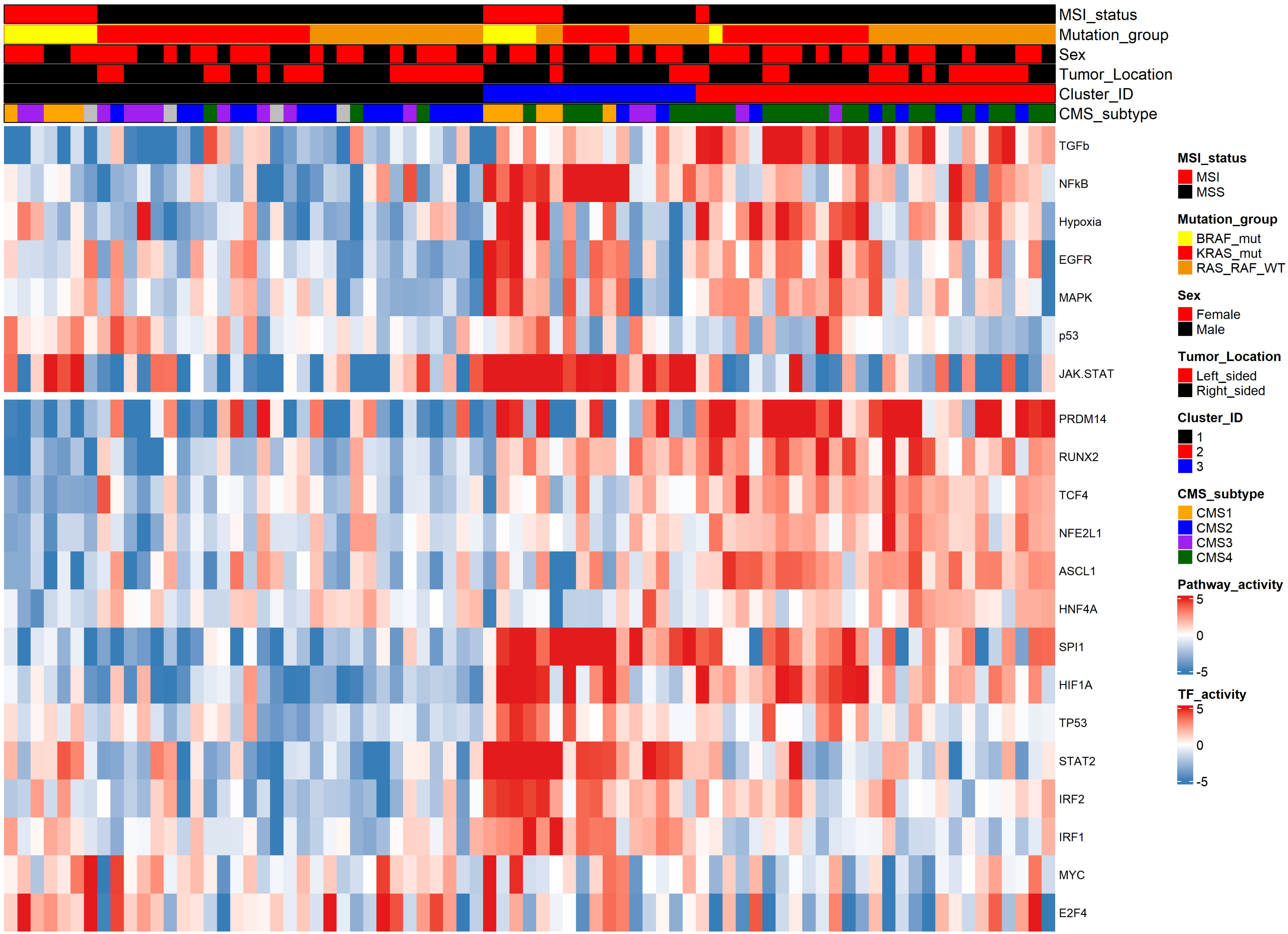

Clustering of the National Cancer Institute's Clinical Proteomic Tumor Analysis Consortium Colon Adenocarcinoma (CPTAC-COAD) data-set based on transcriptomic profiling. Normalized enrichment scores (NES) for the common 7 pathways and 14 transcription factor (TF) activities (see Fig. 3) were employed for unsupervised clustering via principal component analysis (HCPC) using the R packages FactoMineR (version 2.8) and Factoshiny (version 2.4; http://factominer.free.fr/graphs/factoshiny.html). This framework classified the CPTAC-COAD samples into 3 distinct groups. Hierarchical clustering ("complete" linkage, "euclidean" distance) was applied within each pathway and TF sub-cluster, organizing samples by cluster ID, and subsequently based on microsatellite instability (MSI) status and mutational landscape. NES values for pathway and TF activities are depicted as red (higher activity) to blue (lower activity) gradients.
